# Prevalence characteristics of cervical human papillomavirus (HPV) infection in the Zhoupu District, Shanghai City, China

**DOI:** 10.1186/s12985-020-01352-8

**Published:** 2020-06-26

**Authors:** Huaping Li, Peiqun Li, Luyi Huang, Liping Sun, He Ren, Ping Li

**Affiliations:** 1 Shanghai University of Medicine & Health Sciences Affiliated Zhoupu Hospital, Shanghai, China; 2 Renji Hospital Affiliated to Shanghai Jiao Tong University, Shanghai, China; 3 Fengcheng Community Healthcare Center, Shanghai, China; 4 Shanghai University of Medicine & Health Sciences, Shanghai, China

**Keywords:** Age-specific, Prevalence rate, Trend, HPV, China

## Abstract

**Background:**

Human papillomavirus (HPV) infection is the leading cause of genital diseases. It can cause a series of cervical lesions. The distribution of HPV genotypes indicates that the increased prevalence of high-risk HPV (HR-HPV) is positively correlated with the severity of cervical lesions. In addition, persistent HR-HPV infection is associated with the risk of cervical cancer. Considering the latest approval of homemade HPV vaccine in China and the prevalence of HPV distribution, this is of great significance for guiding HPV vaccination work.

**Objective:**

Our study’s purpose was to examine trends of cervical HPV infection rate in each 5-year age group from 2011 to 2019.

**Methods:**

Retrospective analysis of human papillomavirus prevalence rate of 59,541 women from 2011 to 2019 in the District Zhoupu of Shanghai City in China. HPV genotype testing is performed using a commercial kit designed to detect 15 high-risk HPV genotypes and 6 low-risk HPV genotypes. Trends were examined for each 5-year age group.

**Results:**

In the District Zhoupu of Shanghai City in China, the prevalence rate of cervical HPV increased significantly among women aged 15–34 years. The most prevalent HR-HPV genotypes were 52, 16, 58, 53, 39, and 51.

**Conclusion:**

Cervical HPV prevalence rate is very high in younger women in suburb Shanghai. Due to significant differences in infection rates between specific age groups and HPV subtypes, timely intervention is required for these vulnerable populations.

## Introduction

Cervical cancer is the fourth most common cancer among women worldwide and the fourth leading cause of cancer-related deaths [1]. Persistent human papillomavirus (HPV) infection, especially high-risk HPV (HR-HPV) infection, is the main cause of precancerous lesions and cervical cancer [2, 3], and is closely related to cervical and reproductive tract cancers [4]. However, the prevalence of HPV infection remains at a high level in China. In China alone, it is estimated that 98,900 cervical cancer cases, accounting for approximately 20% of total new cases globally, and nearly 30,500 cervical cancer deaths occur each year [5].

A meta-analysis made by Li et al. showed that the overall infection rate of HR-HPV in mainland Chinese women was 19.0% [6] and the national overall prevalence of HPV infection was 15.54% [7]. Some studies have investigated the HPV infection rate and the genotype distribution of Shanghai China in Minhang district [8], female [9] and male [10] patients in the Songjiang district. Our study’s purpose was to examine trends in HPV prevalence from 2011 to 2019 in the District Zhoupu of Shanghai City in China, among each 5- year age group, comparing prevalence rates.

## Methods

### Data source

We extracted 59,541 women data on HPV prevalence in China at Shanghai Zhoupu Hospital from 2011 to 2019. The data is in the form of 5-year age groups. As a regional medical center, Shanghai Zhoupu Hospital provides medical and health services for about 500,000 people in nearby districts and towns.

### Ethics statement

This study was approved by the Institutional Medical Ethics Review Board of Zhoupu Hospital in Shanghai City. The participant received informed consent. For participants under the age of 18, the parents signed a consent form. Confidentiality was ensured during the data collection process at Zhoupu Hospital. Data is analyzed anonymously.

### HPV genotyping

By using Human papillomavirus (HPV) typing test kits (PCR + membrane hybridization) analysis, HPV genotyping was performed on the collected specimens. This assay has been approved by the Chinese FDA (Certification Number (2014): 3402188).

PCR membrane hybridization was used to detect 21 HPV genotypes (6,11,16,18,31,33,35,39,42,43,44,45,51,52,56,58,59,66,68 and 81) by reverse dot hybridization and envelope specific probe membrane hybridization. Sample collection and DNA extraction were the same as PCR fluorescence method. The PCR mix, Taq enzyme and DNA template were mixed in proportion with the typing test kit provided by China Chaozhou Hybribio biochemistry Co., Ltd. (Stock code: 300639.SZ). Amplification parameters were: 95 °C 9 min, 40 cycles (95 °C 20s, 55 °C 30s, 72 °C 30s), 72 °C 5 min. All outcomes were used in the hybridization process. After color rendering, the positive test results were clear blue and purple dots. According to the distribution of HPV types in membrane strips, the positive points were determined. Negative and positive control were set up in the whole process. Internal quality control and external quality assessment was taken in the experiment and the results met the requirements.

### Statistical analysis

All HPV data from 2011 to 2019 were entered into an Excel spreadsheet and then analyzed on the R platform (www.r-project.org) (v3.2.0) and R packages, and the overall and type-specific prevalence of HPV were calculated. All genotypes from single and multiple infections were computed individually. These data were also stratified by age(< 20 years, 20–24 years, 25–29 years,30–34 years, 35–39 years,40–44 years, 45–49 years, 50–54 years, 55–59 years, 60–64 years, 65–69 years,70–74 years, 75–79, years 80–84 years, ≥85 years). The HPV prevalence rate was estimated by a proportion and summarized as a percentage. The secular trends for HPV subtypes and for HPV infection rates of different ages were calculated using student t-test. *p* < 0.05 was considered statistically significant.

## Results

### Characteristics of the study participants

In this study, a total of 59,541 women from Shanghai Zhoupu Hospital, underwent outpatient gynecological examinations and met the participation criteria. The ages of the participants were between 15 and 94 years. The mean age was 37.49. Among the 59,541 subjects, 10,670 women were positive for HPV infection, with a total HPV infection rate of 17.92% (10,670 / 59,541) and a HR-HPV positive rate of 15.56% (9263 / 59,541). Therefore, 86.81% of the infections were caused by HR-HPV.

The six most prevalent HR-HPV genotypes were HPV 52, 16, 58, 53, 39, and 51; however, the top three LR-HPV genotypes were HPV 81,6 and 11. (Table 1).

**Table 1:** Single and multiple type infection rates of different HPV subtypes in District Zhoupu of Shanghai City

HPV subtype	Positive, n(%)	Single-type infection, n(%)	Multiple-type infection, n (%)
HPV52	2130 (3.58)	1226 (2.06)	904 (1.52)
HPV16	1695 (2.85)	1008 (1.69)	687 (1.15)
HPV58	1571 (2.64)	822 (1.38)	749 (1.26)
HPV81	1155 (1.94)	603 (1.01)	552 (0.93)
HPV53	1080 (1.81)	551 (0.93)	529 (0.89)
HPV39	871 (1.46)	448 (0.75)	423 (0.71)
HPV51	863 (1.45)	448 (0.75)	415 (0.7)
HPV33	762 (1.28)	333 (0.56)	429 (0.72)
HPV6	636 (1.07)	293 (0.49)	343 (0.58)
HPV18	603 (1.01)	302 (0.51)	301 (0.51)
HPV68	590 (0.99)	305 (0.51)	285 (0.48)
HPV31	575 (0.97)	275 (0.46)	300 (0.5)
HPV11	542 (0.91)	249 (0.42)	293 (0.49)
HPV66	494 (0.83)	220 (0.37)	274 (0.46)
HPV56	340 (0.57)	161 (0.27)	179 (0.3)
HPV59	238 (0.4)	99 (0.17)	139 (0.23)
HPV44	207 (0.35)	107 (0.18)	100 (0.17)
HPV35	173 (0.29)	80 (0.13)	93 (0.16)
HPV45	173 (0.29)	85 (0.14)	88 (0.15)
HPV42	137 (0.23)	76 (0.13)	61 (0.1)
HPV43	66 (0.11)	26 (0.04)	40 (0.07)

### Trends of different HPV subtype infection rates

Among these 21 HPV subtypes examined, HPV 52, 16, and 58 were also most commonly seen in women both with single-type and multiple-type HPV infections (Table 1). Here, HPV subtype infection rate is calculated as the ratio of the number of HPV subtype infections to the total number of participants in this study (*N* = 59,541).

We investigated the temporal trend of HPV subtype infection. From 2011 to 2019, HPV16, 52, and 58 infection rates were higher. Except for HPV43, there are significant differences in secular trends for most HPV subtypes from 2011 to 2019 (*P* < 0.05, Table 2).

**Table 2:** Secular trends of different HPV subtype infection rates from 2011 to 2019

HPV subtype	2011,n(%)	2012,n(%)	2013,n(%)	2014,n(%)	2015,n(%)	2016,n(%)	2017,n(%)	2018,n(%)	2019,n(%)	*P* value for trend
HPV16	58 (17.52)	107 (13.21)	181 (14.62)	216 (10.34)	240 (10.95)	251 (10.87)	378 (10.99)	263 (10.59)	1 (11.11)	0.002
HPV52	57 (17.22)	118 (14.57)	178 (14.38)	296 (14.18)	317 (14.46)	286 (12.38)	482 (14.01)	393 (15.83)	3 (33.33)	0.0029
HPV58	29 (8.76)	110 (13.58)	114 (9.21)	228 (10.92)	202 (9.22)	258 (11.17)	343 (9.97)	286 (11.52)	1 (11.11)	0.003
HPV81	23 (6.95)	58 (7.16)	97 (7.84)	177 (8.48)	203 (9.26)	225 (9.74)	234 (6.8)	137 (5.52)	1 (11.11)	0.0033
HPV53	21 (6.34)	58 (7.16)	75 (6.06)	139 (6.66)	164 (7.48)	181 (7.84)	243 (7.06)	199 (8.01)	0 (0)	0.0012
HPV11	21 (6.34)	39 (4.81)	64 (5.17)	77 (3.69)	82 (3.74)	67 (2.9)	112 (3.26)	80 (3.22)	0 (0)	0.0037
HPV18	19 (5.74)	40 (4.94)	54 (4.36)	79 (3.78)	96 (4.38)	77 (3.33)	133 (3.87)	105 (4.23)	0 (0)	0.0022
HPV6	17 (5.14)	44 (5.43)	52 (4.2)	105 (5.03)	105 (4.79)	104 (4.5)	118 (3.43)	91 (3.66)	0 (0)	0.002
HPV31	15 (4.53)	38 (4.69)	68 (5.49)	101 (4.84)	72 (3.28)	93 (4.03)	122 (3.55)	66 (2.66)	0 (0)	0.0022
HPV33	15 (4.53)	68 (8.4)	67 (5.41)	139 (6.66)	108 (4.93)	117 (5.06)	148 (4.3)	100 (4.03)	0 (0)	0.0019
HPV66	13 (3.93)	18 (2.22)	37 (2.99)	54 (2.59)	76 (3.47)	82 (3.55)	118 (3.43)	96 (3.87)	0 (0)	0.0047
HPV68	12 (3.63)	26 (3.21)	70 (5.65)	89 (4.26)	77 (3.51)	81 (3.51)	133 (3.87)	102 (4.11)	0 (0)	0.0031
HPV39	7 (2.11)	26 (3.21)	41 (3.31)	112 (5.36)	151 (6.89)	166 (7.19)	212 (6.16)	156 (6.28)	0 (0)	0.0076
HPV45	5 (1.51)	7 (0.86)	20 (1.62)	29 (1.39)	17 (0.78)	30 (1.3)	43 (1.25)	20 (0.81)	2 (22.22)	0.0033
HPV56	5 (1.51)	20 (2.47)	36 (2.91)	41 (1.96)	44 (2.01)	37 (1.6)	98 (2.85)	59 (2.38)	0 (0)	0.0056
HPV51	4 (1.21)	7 (0.86)	18 (1.45)	105 (5.03)	155 (7.07)	151 (6.54)	257 (7.47)	166 (6.69)	0 (0)	0.016
HPV59	4 (1.21)	10 (1.23)	24 (1.94)	31 (1.48)	33 (1.51)	38 (1.65)	53 (1.54)	45 (1.81)	0 (0)	0.0032
HPV35	3 (0.91)	11 (1.36)	12 (0.97)	31 (1.48)	20 (0.91)	20 (0.87)	44 (1.28)	32 (1.29)	0 (0)	0.0047
HPV44	2 (0.6)	2 (0.25)	14 (1.13)	26 (1.25)	19 (0.87)	28 (1.21)	69 (2.01)	47 (1.89)	0 (0)	0.0182
HPV42	1 (0.3)	2 (0.25)	14 (1.13)	9 (0.43)	10 (0.46)	10 (0.43)	62 (1.8)	29 (1.17)	0 (0)	0.0488
HPV43	0 (0)	1 (0.12)	2 (0.16)	4 (0.19)	1 (0.05)	8 (0.35)	38 (1.1)	11 (0.44)	1 (11.11)	0.0868
Total	331	810	1238	2088	2192	2310	3440	2483	9	

### Trends of single-type and multiple-type HPV infection rates

Single HPV genotype infection was found to be the most common pattern (72.32%, 7717/10,670). The multiple genotype infection rate was 27.68% (2953/10,670). Single-type infection (72.32%) was more common than multiple-type infection (27.68%). And the most mixed infection included 9 genotypes(Table 3).

**Table 3:** Single-type and Multiple-type HPV infection rate in District Zhoupu of Shanghai City

Number (HPV subtype)	Infection	Infection Rate(%)
1 HPV subtype	7717	72.32
2 HPV subtypes	2065	19.35
3 HPV subtypes	610	5.72
4 HPV subtypes	203	1.9
5 HPV subtypes	51	0.48
6 HPV subtypes	13	0.12
7 HPV subtypes	9	0.08
8 HPV subtypes	1	0.01
9 HPV subtypes	1	0.01

Secular trends of the number of HPV single-type infection and multiple-type infection were also calculated. We found that the HPV numbers of single-type infection and some of multiple-type (2–5,7) infection rates among women showed significant differences from 2011 to 2019 (all *P* < 0.05, Table 4).

**Table 4:** Secular trends in single-type and multiple-type HPV infection rates from 2011 to 2019

Number of (HPV infection subtypes)	2011,n(%)	2012,n(%)	2013,n(%)	2014,n(%)	2015,n(%)	2016,n(%)	2017,n(%)	2018,n(%)	2019,n(%)	*P* value for trend
1 HPV subtypes	160 (69.87)	417 (71.28)	600 (70.18)	1040 (70.51)	1119 (71.78)	1235 (73.91)	1737 (71.48)	1400 (75.39)	9 (100)	0.0034
2 HPV subtypes	46 (20.09)	128 (21.88)	173 (20.23)	309 (20.95)	312 (20.01)	291 (17.41)	478 (19.67)	328 (17.66)	0 (0)	0.0029
3 HPV subtypes	14 (6.11)	29 (4.96)	53 (6.2)	92 (6.24)	82 (5.26)	96 (5.75)	146 (6.01)	98 (5.28)	0 (0)	0.0036
4 HPV subtypes	8 (3.49)	6 (1.03)	19 (2.22)	22 (1.49)	32 (2.05)	42 (2.51)	50 (2.06)	24 (1.29)	0 (0)	0.0048
5 HPV subtypes	0 (0)	4 (0.68)	6 (0.7)	8 (0.54)	11 (0.71)	6 (0.36)	10 (0.41)	6 (0.32)	0 (0)	0.0033
6 HPV subtypes	1 (0.44)	1 (0.17)	2 (0.23)	2 (0.14)	1 (0.06)	0 (0)	6 (0.25)	0 (0)	0 (0)	0.0512
7 HPV subtypes	0 (0)	0 (0)	1 (0.12)	2 (0.14)	2 (0.13)	1 (0.06)	2 (0.08)	1 (0.05)	0 (0)	0.01
8 HPV subtypes	0 (0)	0 (0)	1 (0.12)	0 (0)	0 (0)	0 (0)	0 (0)	0 (0)	0 (0)	0.3434
9 HPV subtypes	0 (0)	0 (0)	0 (0)	0 (0)	0 (0)	0 (0)	1 (0.04)	0 (0)	0 (0)	0.3434
Total	462	1214	1794	2976	3218	3450	4928	3796	19	0

### Trend analysis stratified by 5-year age groups from 2011 to 2019

**Fig. 1:**
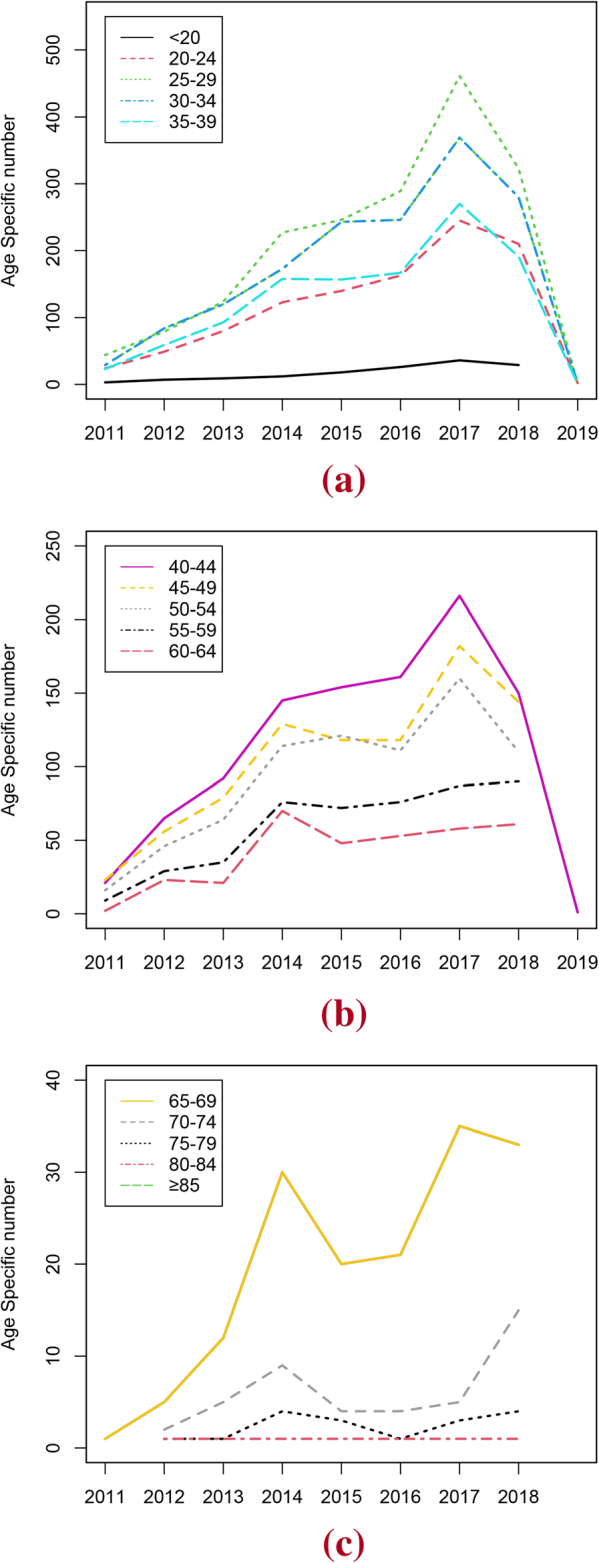
Trend analysis stratified by 5-Year Age groups from 2011 to 2019. **a** prevalence numbers of women (< 20, 20–24,25-29,30-34,35–39) **b** prevalence numbers of women (40–44,45-49,50-54,55-59,60–64) **c** prevalence numbers of women (65–69,70-74,75-79,80–84,≥85)

For women aged < 55 years, the highest prevalence numbers occurred in 2017, and the prevalence rate decreased from then. Prevalence number of HPV was significantly higher in women aged 25–34 years in Fig. 1a, in contrast to women older than 65 years in Fig. 1c.

Secular trends of HPV infection rates of different ages were also calculated. We found that HPV positive rates among women aged < 80 years also showed significant differences from 2011 to 2019 (all *P* < 0.05, Table 5).

**Table 5:** Secular trends of HPV infection rates of different ages from 2011 to 2019

Age (years)	2011,n(%)	2012,n(%)	2013,n(%)	2014,n(%)	2015,n(%)	2016,n(%)	2017,n(%)	2018,n(%)	2019,n(%)	*P* value for trend
< 20	5 (1.08)	9 (0.74)	14 (0.78)	17 (0.57)	28 (0.87)	32 (0.93)	41 (0.83)	31 (0.82)	0 (0)	0.0029
20–24	31 (6.71)	64 (5.27)	96 (5.35)	156 (5.24)	174 (5.41)	192 (5.57)	278 (5.64)	249 (6.56)	2 (11.11)	0.0026
25–29	51 (11.04)	93 (7.66)	151 (8.42)	268 (9.01)	301 (9.35)	352 (10.2)	546 (11.08)	384 (10.12)	1 (5.56)	0.0039
30–34	34 (7.36)	101 (8.32)	145 (8.08)	201 (6.75)	285 (8.86)	313 (9.07)	422 (8.56)	329 (8.67)	2 (11.11)	0.0028
35–39	28 (6.06)	69 (5.68)	116 (6.47)	176 (5.91)	189 (5.87)	198 (5.74)	320 (6.49)	211 (5.56)	1 (5.56)	0.0026
40–44	28 (6.06)	78 (6.43)	112 (6.24)	165 (5.54)	184 (5.72)	183 (5.3)	245 (4.97)	171 (4.5)	1 (5.56)	0.0013
45–49	26 (5.63)	69 (5.68)	92 (5.13)	157 (5.28)	141 (4.38)	143 (4.14)	215 (4.36)	169 (4.45)	0 (0)	0.0014
50–54+	16 (3.46)	52 (4.28)	76 (4.24)	138 (4.64)	136 (4.23)	132 (3.83)	187 (3.79)	126 (3.32)	1 (5.56)	0.0018
55–59	9 (1.95)	34 (2.8)	47 (2.62)	85 (2.86)	88 (2.73)	91 (2.64)	95 (1.93)	105 (2.77)	1 (5.56)	0.0016
60–64	2 (0.43)	28 (2.31)	27 (1.51)	79 (2.65)	52 (1.62)	59 (1.71)	66 (1.34)	67 (1.77)	0 (0)	0.0024
65–69	1 (0.22)	6 (0.49)	13 (0.72)	33 (1.11)	22 (0.68)	23 (0.67)	38 (0.77)	35 (0.92)	0 (0)	0.0047
70–74	0 (0)	2 (0.16)	6 (0.33)	9 (0.3)	5 (0.16)	6 (0.17)	6 (0.12)	16 (0.42)	0 (0)	0.0099
75–79	0 (0)	1 (0.08)	2 (0.11)	4 (0.13)	4 (0.12)	1 (0.03)	3 (0.06)	4 (0.11)	0 (0)	0.0057
80–84	0 (0)	1 (0.08)	0 (0)	0 (0)	0 (0)	0 (0)	1 (0.02)	1 (0.03)	0 (0)	0.0805
≥85	0 (0)	0 (0)	0 (0)	0 (0)	0 (0)	0 (0)	1 (0.02)	0 (0)	0 (0)	0.3466
Total	462	1214	1794	2976	3218	3450	4928	3796	18	0

## Discussion

Our study analyzed HPV prevalence trends of Zhoupu district in Shanghai city from 2011 to 2019. While the overall trend demonstrated there was an increase in prevalence rate, women aged < 55 years in 2017 experienced the highest infection numbers, and a decline in HPV infection rate from 2018 to 2019. Zhoupu district is located in the suburb of Pudong New Area, Shanghai City of China. According to the analysis report of Pudong’s population development in 2017 issued by the development and Reform Commission of Pudong New Area Government in Shanghai, there is a significant increase in the population development situation. So, the number of patients seeking medical treatment in the Zhoupu area also increases. The main reasons may be as follows: first, with the implementation of the two-children policy, the number and proportion of the two children have increased steadily; second, a large number of floating populations have gathered in the suburbs; third, the population ageing has deepened.

Single-type HPV infection and some of multiple-type (2–5,7) infection rates among women showed significant differences. HPV prevalence trends stratified by 5-year age groups demonstrated that HPV positive rates among women aged < 80 years also showed significant differences.

In the 59,541 outpatient women, the overall prevalence of HPV was 17.92% and the HR-HPV prevalence was 15.56%. Compared with similar studies in China, the prevalence in our study was higher than that in Xinjiang (14.02%) [11], Urumqi (16.74%) [12], Zhejiang (17.6%) [13], and Nanjing KingMed Diagnostics (17.7%) [14], but lower than that in Meizhou (18.34%) [15], Wuhan (18.6%) [13], Beijing (20.16%) [4], Guangdong (21.06%) [16] Shandong (28.4%) [17], and three cities (Xuzhou, Nanjing and Suzhou) of Jiangsu Province (26.92%) [18]. Different HPV prevalence rates may be a combination of different economic levels, cultural diversity, geographic locations, and survey periods.

Analysis of HPV prevalence indicates that the most common HR-HPV genotypes in outpatient women are HPV 52,16,58,53 and 39. These are the same common genotypes as Meizhou [15]. This is similar to other studies conducted in China. HPV 16,58, 52, 53, and 39 were the dominant subtypes in Jiangsu [14]. HPV 52,16 and 58 had a higher prevalence in Beijing [4]. HPV 52, 16, 58, CP8304, and 53 were the dominant subtypes among gynecological outpatients among women in Guangdong, China 2008 to 2017 [16]. The most common HR-HPV group included HPV types 16, 31, 33, 35, 52, and 58 in Zhejiang and Wuhan [13]. HPV 16, 52, 58, 51, and 56 were the five most common HR-HPV genotypes in Shandong [17]. The most prevalent genotypes were HPV 16, 52,58,53 and 31 in Xinjiang [11]. From the analysis of 303 China-specific articles, the top 4 common HPV types were detected HPV 16, 18, 58, 52 in descending order of frequency [7]. The top 5 subtypes with the highest infection rates were 16, 52, 58, 53, and 18 in a systematic review of the epidemiology of HR-HPV infections in mainland Chinese women from January 2000 to June 2018 [6]. Among all these studies above, HPV 52, 16 and 58 were the most common subtypes found, so vaccines against HPV 52, 16 and 58 should be developed for Chinese women.

Our research shows that HPV prevalence of women in Zhoupu District of Shanghai is age-related and single-type was more common than multiple-type HPV infection. The same is true in the rest of China, such as Guangdong and Beijing [4, 16]. Single-type was more common; however, multiple-type HPV infections are more dangerous than single-type infections. The HPV infection reached a peak in women aged 25–29 years. Perhaps because women in this age group are more sexually active and have more than one sexual partner, so HPV infection rates are higher than in other age groups. Our study may provide valuable data to inform cervical cancer screening and HPV vaccination programs for women in Shanghai.

Our research has an important policy implication: it is necessary to vaccinate Chinese women nationwide, especially young women, against HPV infection. Injecting HPV vaccine can induce a durable anti-HPV response and prevent HPV infection. At present, China has 2- and 4-valent HPV vaccines, and 9-valent vaccines in some regions (since May 2018). However, the proportion of women who are willing to take the initiative to receive the HPV vaccine is still very low, and various measures are urgently needed to increase the vaccination rate in China.

Our research focuses on the prevalence of HPV in different HPV-subtype-related and age-related groups, which allowed us to identify trends in differences in 2011–2019. However, our study also has some limitations. Although Zhoupu Hospital is the largest public hospital in the local area, it is located in the suburbs. These areas have large population mobility and relatively poor data quality.

In the future, we plan to examine the relationship between the mortality rate of cervical cancer and the HPV prevalence in Shanghai Zhoupu district. And we will compare it with the two databases from the World Health Organization Cancer Mortality Database (International Agency for Research on Cancer, Lyon, France) and China Health Statistical Yearbooks, like what Min did [5].

## Conclusions

In conclusion, we conducted a population-based study on the prevalence of HPV-subtype-related and age-related groups in the Zhoupu area of Shanghai. The cervical HPV prevalence rate is very high in younger women aged 25–34 years in suburb Shanghai city. The prevention of HPV-related diseases is challenging. There are significant differences in infection rates between specific age groups and HPV subtypes, and this finding will provide guidance for future vaccination programs and improve their accuracy.

## Data Availability

The data were collected from Zhoupu Hospital in Shanghai City. We are grateful for their generous help. The data can be freely shared. The materials were purchased from Hybribio Biotechnology Limited Corp.

## Abbreviations

HPV: Human papillomavirus
(HR-HPV): High-risk HPV
